# An Unbiased Flow Cytometry-Based Approach to Assess Subset-Specific Circulating Monocyte Activation and Cytokine Profile in Whole Blood

**DOI:** 10.3389/fimmu.2021.641224

**Published:** 2021-04-26

**Authors:** Jurij Kiefer, Johannes Zeller, Balázs Bogner, Isabel A. Hörbrand, Friederike Lang, Emil Deiss, Oscar Winninger, Mark Fricke, Sheena Kreuzaler, Eva Smudde, Markus Huber-Lang, Karlheinz Peter, Kevin J. Woollard, Steffen U. Eisenhardt

**Affiliations:** ^1^ Department of Plastic and Hand Surgery, Medical Center – University of Freiburg, Medical Faculty of the University of Freiburg, Freiburg, Germany; ^2^ Department of Traumatology, Hand, Plastic, and Reconstructive Surgery, Center of Surgery, University of Ulm, Ulm, Germany; ^3^ Atherothrombosis and Vascular Biology, Department of Cardiometabolic Health, Baker IDI Heart and Diabetes Institute, Melbourne, VIC, Australia; ^4^ Centre of Inflammatory Disease, Imperial College London, London, United Kingdom

**Keywords:** inflammation, blood monocytes, flow cytometry, cell activation, cytokines

## Abstract

Monocytes are the third most frequent type of leukocytes in humans, linking innate and adaptive immunity and are critical drivers in many inflammatory diseases. Based on the differential expression of surface antigens, three monocytic subpopulations have been suggested in humans and two in rats with varying inflammatory and phenotype characteristics. Potential intervention strategies that aim to manipulate these cells require an in-depth understanding of monocyte behavior under different conditions. However, monocytes are highly sensitive to their specific activation state and expression of surface markers, which can change during cell isolation and purification. Thus, there is an urgent need for an unbiased functional analysis of activation in monocyte subtypes, which is not affected by the isolation procedure. Here, we present a flow cytometry-based protocol for evaluating subset-specific activation and cytokine expression of circulating blood monocytes both in humans and rats using small whole blood samples (50 - 100 μL). In contrast to previously described monocyte isolation and flow cytometry visualization methods, the presented approach virtually leaves monocyte subsets in a resting state or fixes them in their current state and allows for an unbiased functional endpoint analysis without prior cell isolation. This protocol is a comprehensive tool for studying differential monocyte regulation in the inflammatory and allogeneic immune response *in vitro* and *vivo*.

## Introduction

Monocytes are white blood cells of the mononuclear phagocyte system, which derive from precursors in the bone marrow and circulate in the blood with a half-life of 1- 2 days before migrating into various tissues to replenish macrophages (1). Blood monocytes are involved in the innate response to bacterial, fungal, parasitic, and viral infection (2). Thus, monocytes are the immune system’s cornerstones linking innate and adaptive immunity and are critical drivers in many inflammatory diseases. While monocytes and macrophages were almost universally considered two related cell types that arise from a continuum of differentiation, recent studies have challenged this dogma’s generalized applicability based on three key findings. First, monocytes do not substantially contribute to most tissue macrophage compartments in the steady-state or certain inflammation types. Second, mature tissue macrophages are derived from embryonic precursors that seed the tissues before birth. And third, tissue macrophages can maintain themselves in adults by self-renewal (3).

By means of flow cytometry, three monocytic subpopulations have been suggested in humans based on the differential expression of the pattern recognition receptor for lipopolysaccharide CD14 and the low-affinity IgG receptor CD16 (FcγIII), as CD14^++^ [CD14^++^ CD16^-^, classical], CD14^++^ CD16^+^ [intermediate], and CD14^dim^ [CD14^+^ CD16^++^, non-classical] monocytes (4). The CD14^++^ monocytes represent about 85% of circulating monocytes under physiological conditions. As classical monocytes are mainly involved in phagocytosis, they can be recruited quickly to sites of infection. The remaining 15% consists of CD14^dim^ and CD14^++^CD16^+^ monocytes, which predominantly unfold pro-inflammatory characteristics (5–9). The number of CD16-positive monocytes can increase or decrease strongly under various conditions. Studies have suggested dynamic capacities for monocytes with the sequential transition of CD14^++^ monocytes towards intermediate and non-classical forms in inflammation and homeostasis. For instance, high levels of CD16^+^ monocytes have been reported for malignancy and many inflammatory conditions, with an increase to more than 50% of all monocytes for severe infections like sepsis (5, 10–12).

Differentiation of monocyte subsets based only on quantification of surface CD14 and CD16 expression fails to correctly distinguish monocytes from other leukocyte subsets, such as CD16-expressing neutrophil granulocytes and natural killer cells (13). Thus, different staining protocols using either CD68, CD86, or HLA-DR as the third pan-monocytic marker have been published (14–16). Furthermore, different protocols have highlighted that monocyte isolation from whole blood can profoundly affect the plasticity of the cells (17, 18), which impedes the assessment of monocyte activation. Monocyte subsets are susceptible to their activation state, and, thus, surface expression markers can change in the course of cell isolation. To identify and quantify monocyte activation, Eisenhardt et al. developed a single-chain antibody (scFv, MAN-1) directed against activation-specific macrophage antigen-1 ligand-binding sites (19). The macrophage antigen-1 (Mac-1, CD11b/CD18, integrin α_M_β_2_) is expressed constitutively on the surface of neutrophils and monocytes and plays a pivotal role in inflammation and host defense. Mac-1 undergoes a conformational change upon leukocyte activation, exposing interaction sites for multiple ligands, such as fibrinogen, heparin (20), or iC3b (19). Therefore, the CD11b/CD18 receptor’s conformational change is a surrogate for monocyte activation.

In rats, monocyte subsets have been defined based on high or low CD43 expression with CD43^++^ cells demonstrating phenotypically high levels of CD4 (1, 21). Infusion of IFNγ into rats has led to a substantial increase of CD43^++^ CD4^low^ monocytes (22). The CD43^++^ monocytes have also been shown to be low for C-C chemokine receptor type 2 (CCR2), high for CXC3 chemokine receptor type 1 (CX3CR1) (23), and low to negative for CD62L. While limited, this evidence suggests that the CD43^++^ rat monocytes are homologous to the non-classical monocytes in humans. Optimized staining protocols implemented the granulocyte and erythroid cell antibody clone HIS48 and signal regulatory protein α (CD172a) for discrimination of monocyte subpopulations (24). However, monocyte subset isolation in rats tends to affect cell distribution to a greater extent than in humans due to cells being more sensitive to standard lysing solutions (21).

Here, we present a flow cytometry-based protocol to accurately evaluate subset-specific activation and cytokine expression in circulating blood monocytes both in humans and rats. Cell isolation and purification can affect subset distribution (18), lead to pre-activation of monocytes (17), and, in turn, confound experimental results. In contrast, our functional endpoint analysis allows for a non-confounded detection of subset-specific cell activity in small whole blood samples without prior cell isolation. We also put ready-to-use lysis solutions to the test as we analyze the monocyte activation state by detecting the conformational change of the CD11b/CD18 receptor (macrophage-1 antigen, Mac-1). The presented protocol is a comprehensive tool for studying differential monocyte regulation during the inflammatory and allogeneic immune response, especially in disease models. As little is known about rat monocytes in disease models, the monocyte activation and cytokine expression endpoint analysis can help assess the usefulness of the rat as a convenient animal model for investigating monocyte subsets.

## Materials and Equipment

### Buffers and Antibodies for Analysis of Circulating Monocytes in Humans

RBC lysis buffer (10x):

80.2 g NH_4_Cl
8.4 g NaHCO_3_
126 mL EDTA (100 mM)
1000 mL total end volume, fill up with ddH_2_O
- prepare 1x dilution, preferably 500 mL, and autoclave it before use; keep buffer solution at 4 °C and pH 7.8
- renew buffers every week

FACS buffer (for human and rat analysis):

PBS-BSA (0.5%) 5 g BSA (Sigma: A9418-100G)

 1 L sterile PBS

Fixation buffer (for human and rat analysis):

10x BD CellFix

-prepare 1x Solution with ddH_2_O and store at room temperature

Antibodies:

anti-HLA-DR-FITC (TU36, mouse IgG2b, κ) 1:50
anti-CD14-Pacific Blue^®^ (M5E2, mouse IgG2a, κ) 1:50
anti-CD16-PE-Cy7 (3G8, mouse IgG1, κ) 1:50

Negative lineage markers:

anti-CD2-PE (RPA-2.10, mouse IgG1, κ) 1:50
anti-CD19-PE (HIB19, mouse IgG1, κ) 1:50
anti-CD15-PE (VIMC6, mouse IgM, κ) 1:50
anti-CD56-PE (MY31, mouse IgG2, κ) 1:50
anti-NKp46-PE (BAB281, mouse IgG1, κ) 1:50

### Buffers and Antibodies for Analysis of Circulating Monocytes in Rats

RBC Lysis buffer (10x):

8.3g NH_4_Cl
1g NaHCO_3_
1mL EDTA (100mM)
1000 mL total end volume, fill up with ddH_2_O
-prepare 1x dilution, preferably 500 mL, and autoclave it before use; keep buffer at 4 °C and pH 7.7
-renew buffers every week

Antibodies:

anti-CD172a FITC (OX41 Clone, BIO-RAD #MCA274F) 1:40
anti-CD43 APC (W3/13, BioLegend #202810) 1:40
anti-Granulocyte marker Biotin (HIS48, eBioscience #13-0570-82) 1:40
Streptavidin eFluor 450 (eBioscience #48-4317-82) 1:40
anti-CD11b PE (WT.5, BD Pharmingen #562105) 1:40

All isotype controls were purchased from their respective manufacturers. Stained samples were analyzed using appropriate compensation for correcting spectral overlap and autofluorescence.

## Methods

### Study Subjects

Institutional review board approval was obtained from the local ethical committee prior to data acquisition (ethical approval #112/17). For monocyte subset analysis in humans, ten healthy subjects (five females and five males, aged between 22 and 34 years) volunteered in the study. None of the participants took any medication and had neither fever nor infection in the week before the blood draw.

For monocyte subset analysis in rats, adult male Wistar rats (seven weeks old, Charles River WIGA, Sulzfeld, Germany) served as blood donors. All animals were housed in the animal facility of the Medical Center – University of Freiburg under standard conditions. All procedures were in accordance with the “Principles of Laboratory Animal Care” (NIH publication No. 86-23, revised 1985) as well as the German Law on Animal Protection.

### Blood Sampling and Staining Procedures in Human

According to the guidelines established by the local ethics committee, we obtained all samples after informed consent had been given. We drew fresh whole blood from the median cubital vein of healthy donors, discarded the first 10 mL to avoid unspecific monocyte activation, and collected the remaining blood in heparinized vacutainers (16 IU/mL). As the interval between blood collection and processing is one of the most critical parameters for confounding factors in functional immunological assays (25), we processed all samples within 30 min. For each sample, 100 µL of whole blood was transferred into 2 mL polypropylene collection tubes (Biozym Scientific GmbH). To analyze subset-specific monocyte activation, we then stimulated the blood samples with the reagents under investigation for 15 min in an incubator at 37°C and 5 % CO_2_ in the dark, e.g., 100 ng/mL of lipopolysaccharides (LPS, a TLR4/TLR2 agonist), 500 ng/mL of monophosphoryl lipid A (MPLA, a TLR4 agonist), 50 µg/mL of monomeric C-reactive protein (mCRP), or with the same volume of PBS (vehicle control). CRP is a pentameric protein (pCRP) and marker of inflammation extensively used in clinical practice (26). While circulating pCRP is not pro-inflammatory in healthy subjects, we have previously demonstrated that it exacerbates existing tissue injury in a complement-dependent manner *via* conformation rearrangement to pCRP* (27, 28). We suggested that mCRP partially exerts its pro-inflammatory effect on monocytes *via* CD16-mediated signaling. Since monocyte subsets substantially differ in their CD16 surface expression, we investigated possible subset-specific activation patterns mCRP. Therefore, we used mCRP as a pro-inflammatory factor in our *in vitro* experiments.

Certainly, incubation times for the activation assay may vary depending on the respective study protocol. For disease-related analysis in humans, no stimulation step is required at this point. After stimulation, we immediately added 50 µL of cell fixation solution (BD CellFIX™) at room temperature to all samples and incubated them for 5 min at room temperature (21°C) to stop further cell activation. Then, samples were added 2.4 µL MAN-1 or 2.0 µL of anti-CD11b monoclonal antibody (WT.5, phycoerythrin) and incubated for 15 minutes on ice in the dark to detect activation-dependent integrin upregulation in monocyte subpopulations. Afterward, fixed whole blood was washed with PBS (1:10) to optimize the following erythrocyte lysis.

We performed osmotic lysis of red blood cells using a ratio of 1:20 v/v to an ammonium chloride-based lysis solution (ddH_2_O, NH_4_Cl, NaHCO_3_, EDTA). After lysis and washing, cells were resuspended in flow cytometry buffer solution (PBS, 0.5 % BSA) and stained for 15 mins at 4°C in the dark with anti-human antibodies specific for anti-HLA-DR [TU36, allophycocyanin (APC)], anti-CD14 (M5E2, Pacific Blue^®^), anti-CD16 [3G8, phycoerythrin-cyanine7 (PE-Cy7)], anti-CD2 [RPA-2.10, phycoerythrin (PE)], anti-CD56 (MY31, PE), anti-NKp46 (BAB281, PE), anti-CD15 (VIMC6, PE) and anti-CD19 (J3-119, PE). We then washed and transferred the stained cells to 5 mL FACS tubes (polystyrene) or round-bottomed 96 well plates for immediate analysis. In a second approach, we performed red blood cell lysis using a commercially available ready-to-use RBC lysis buffer (RBC Lysis Buffer, multi-species, eBioscience™; BD FACS™ Lysing solution) according to the manufacturer’s protocol. All antibodies are detailed in **Supplemental Information 1**.

For intracellular detection of cytokines in leukocytes, it is necessary to block cytokines’ secretion by inhibiting protein transport inside the cell. Therefore, we incubated blood samples for 6 hours with the reagents mentioned above in the presence of brefeldin A (BFA, 3 µg/mL) to assess subset-specific cytokine levels in peripheral blood monocytes. Brefeldin A inhibits protein transport from the endoplasmic reticulum to the Golgi complex, allowing intracellular cytokine detection. Thus, this methodological approach detects intracellular cytokine regulation changes within a particular cell population. To assess intracellular cytokine production, blood samples were stimulated in presence of the protein transport inhibitor BFA. After an initial activation period, BFA is added to the stimulated and control blood probes, thus allowing the cells reacting to the pro-inflammatory impact of each reactant. After an appropriate stimulation time (depending on each cytokine to test), we immediately added a cell fixation solution to stop further cytokine production. Then, blood samples were washed, and lysis of red blood cells was performed. After another washing step, cell suspensions were stained in flow cytometry buffer solution with the panel of cell surface markers. Subsequently, we fixed and permeabilized the samples using a commercially available kit (FIX&PERM cell fixation & cell permeabilization kit, ThermoFisher). Then, we added the respective cytokine monoclonal antibody in question, e.g., anti-tumor necrosis factor (TNF; clone Mab11), anti-interleukin 1 beta (IL1b), and anti-interleukin 6 (IL6). Afterward, the cell suspensions were washed before proceeding with flow cytometric analysis.

To analyze the impact of cell isolation procedures on monocyte activity, we evaluate the conformational change of the CD11b/CD18 (MAN-1) receptor in monocytes after cell isolation *via* density gradient centrifugation for isolation of peripheral blood mononuclear cells (PBMC) or fluorescence-activating cell sorting (FACS) for isolation of monocyte subsets. After the respective cell isolation, we stimulated the remaining cell suspensions with PBS (control vehicle), mCRP, and LPS for 15 min as described and added MAN-1 before proceeding with flow cytometric analysis. We then compared the results to our whole blood assay.

Furthermore, we conducted another experiment to compare monocytic cytokine expression assessed by RT-PCR to the results achieved by intracellular cytokine staining. For this purpose, we purified monocytes from PBMC using magnetic-activated cell sorting (MACS; Pan Monocyte Isolation Kit human, Miltenyi Biotec). We then incubated the monocytic cell suspension with PBS (control vehicle) and LPS (10 ng/mL and 100 ng/mL) in 0.5% PBS-BSA for 3, 6, and 24 h in an incubator at 37°C and 5 % CO_2_. After RNA isolation, we accessed the expression of TNF using RT-PCR.

### Blood Sampling and Staining Procedures in Rats

We drew fresh whole blood samples from adult male Wistar rats’ tail vein into heparin-containing collection tubes, mixed them gently, and transferred the cell solution into 2 mL polypropylene collection tubes (100 µL of heparinized whole blood). To analyze subset-specific monocyte activation in rat whole blood, we stimulated cell solution samples and incubated them in the same manner as human samples at 37°C and 5 % CO_2_ in the dark. After stimulation, we immediately added 50 µL of cell fixation solution (BD CellFIX™) to all samples and incubated them for 5 min at room temperature (21°C) to stop further cell activation. For animal disease models, blood samples are drawn at specific time points to evaluate monocytes’ behavior. Thus, no further stimulation step is required for these experiments, and samples are fixed right after blood withdrawal. To detect activation-dependent integrin upregulation of monocyte subpopulations, anti-CD11b (WT.5, FITC) was added as it specifically binds to the α subunit of Mac-1 found on neutrophils and myeloid cells. Afterward, cell solutions were washed in PBS to optimize the following erythrocyte lysis.

We performed osmotic lysis of rat red blood cells using a similar ammonium-based lysis buffer (ddH_2_O, NH_4_Cl, NaHCO_3_, EDTA), which differs in pH to address the more fragile nature of rat monocytes. After lysis and washing, cells were resuspended in flow cytometry buffer solution (PBS, 2 mM EDTA, 0.5 % BSA) and stained for 15 min at 4°C in the dark with anti-rat antibodies specific for anti-CD172a (OX41, APC), anti-CD43 (W3/13, PE), anti-Granulocyte marker (HIS48, biotinylated), and Streptavidin (eFluor 450). Stained cells were then washed and transferred to 5 mL FACS tubes (polystyrene) or round-bottom 96 well plates for immediate analysis. All antibodies are detailed in **Supplemental Information 2**.

To assess subset-specific cytokine expression levels in rat blood monocytes, we incubated, fixed, permeabilized, and stained all samples in the manner mentioned above with the respective monoclonal antibodies for cell surface markers and intracellular cytokines.

### Flow Cytometry Gating Strategy and Data Acquisition

We analyzed samples by flow cytometry (LSR Fortessa, BD Biosciences) utilizing a three-laser-line configuration including blue (488 nm), red (640 nm), and violet (405 nm) laser with standard filter and mirror configuration. For data acquisition, we used the BD FACSDiva software, which automatically calculated compensation matrices for correcting spectral overlap by analyzing single-stained controls. We further evaluated the data using the FlowJo data analysis software (FlowJo, LLC, Ashland, CA).

For human samples, monocytes were first visualized and separated from neutrophil granulocytes and lymphocytes based on size (forward-scattered light, FSC) and granularity/internal complexity (side-scattered light, SSC). An acquisition threshold was set individually to eliminate platelets, dead or pyknotic cells, and debris. Doublets were further on excluded based on FSC area/FSC height and SSC area/SSC width. Then, sequential gating of the remaining cell population against CD14 and CD16 was performed to identify monocyte subsets, separated into large CD14^++^ CD16^-^ and CD14^++^ CD16^+^ monocytes, as well as small CD14^dim^ CD16^++^ monocytes and CD14^-^ CD16^-^ dendritic cells. We further specified monocyte subsets by cells expressing HLA-DR and excluded all cells positive for CD2 (T-cells), CD19 (B-cells), CD56, and NKp46 (lymphocytes and NK-cells, respectively), and CD15 (granulocytes). Lastly, the mean fluorescence intensity (MFI) and the relative number of positive cells for the respective inflammatory marker was measured for each monocyte subpopulation.

For rat samples, monocytes were visualized similarly based on FSC and SSC. An acquisition threshold was set to eliminate unwanted events. Doublets were excluded based on FSC area/FSC height and SSC area/SSC height. Rat monocyte subsets are contained among CD172a^+^ cells, which were plotted against SCC area. Within this gate, murine monocyte subsets were identified using CD43 and HIS48. Subsequently, the MFI of the cell integrin or cytokine under investigation was assessed for each monocyte subpopulation.

### Statistics

The data are presented as mean ± standard error of the mean (SEM) of the mean fluorescence intensity (MFI) or otherwise stated in the text. Monocyte activation and inflammatory markers were compared with baseline measurements of unstimulated cells using analysis of variance with *Tukey post hoc* analysis. Correlations between variables were assessed with the *Pearson* correlation coefficient. *P* values < 0.05 were considered statistically significant. All statistical analyses were performed using GraphPad Prism 9 for Mac (GraphPad Software, LLC, Ltd., La Jolla, CA).

## Results

**Figure 1:**
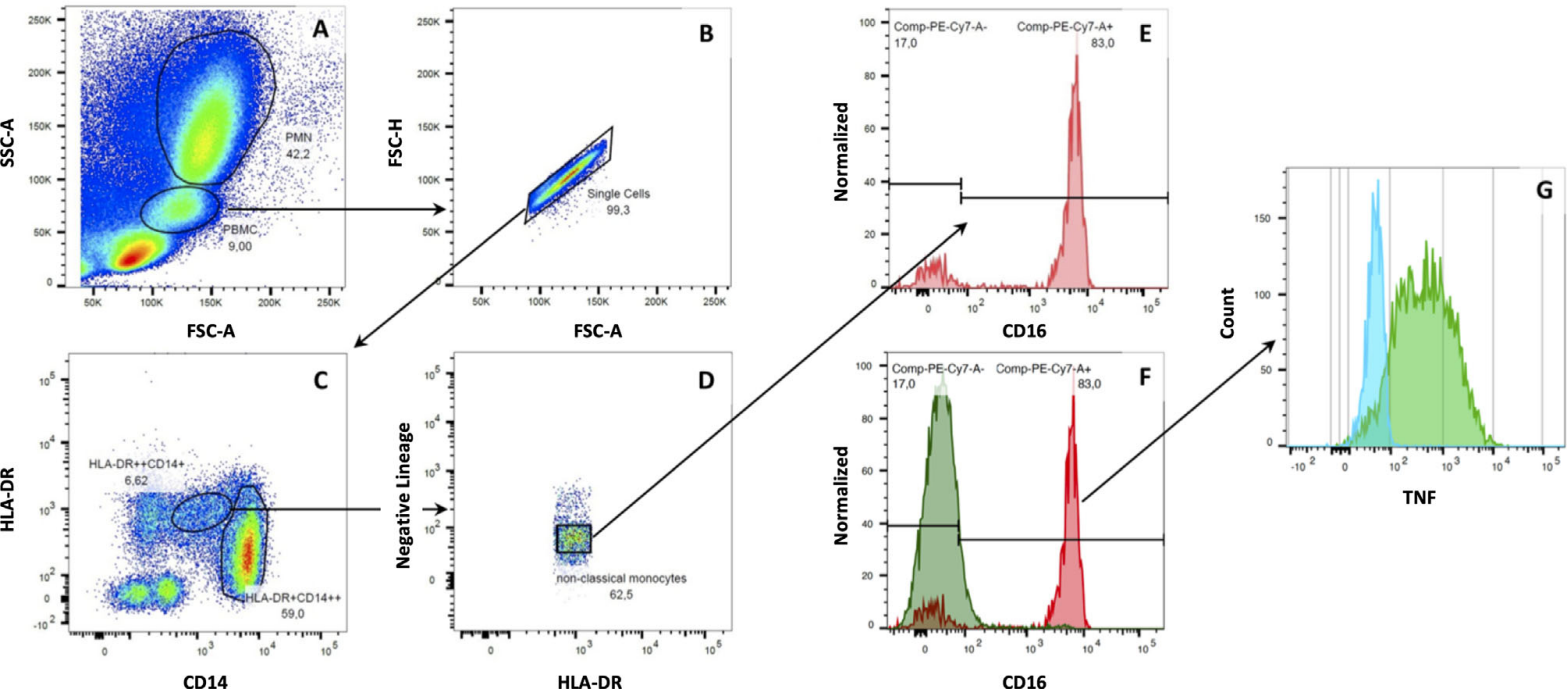
Gating strategy for human monocytes. Monocytes were separated from neutrophil granulocytes and lymphocytes based on cell size and internal complexity. After excluding unwanted events and doublets, monocytes were analyzed amongst CD14^+^ and CD16^+^ cells. Then, monocyte subsets were detected within HLA-DR^+^ cells, which were negative for B-cell (CD19^-^), T-cell (CD2^-^), NK-cell (NKp46^-^ and CD56^-^), or granulocyte (CD15^-^) markers. Here, the following monocytes subpopulations could be identified: classical monocytes (CD14^++^CD16^-^), intermediate monocytes (CD14^++^CD16^+^), and non-classical monocytes (CD14^dim^CD16^++^). Cells without expression of CD14 and CD16 were considered dendritic cells of the peripheral blood **(A–D)**. After incubating human whole blood samples with LPS and mCRP for six hours, we found a down-regulation of CD16 on non-classical monocytes. Non-classical monocytes had to be backtracked within the HLA-DR^+^ gate to assess their specific cytokine expression **(E–G)**.

**Figure 2:**
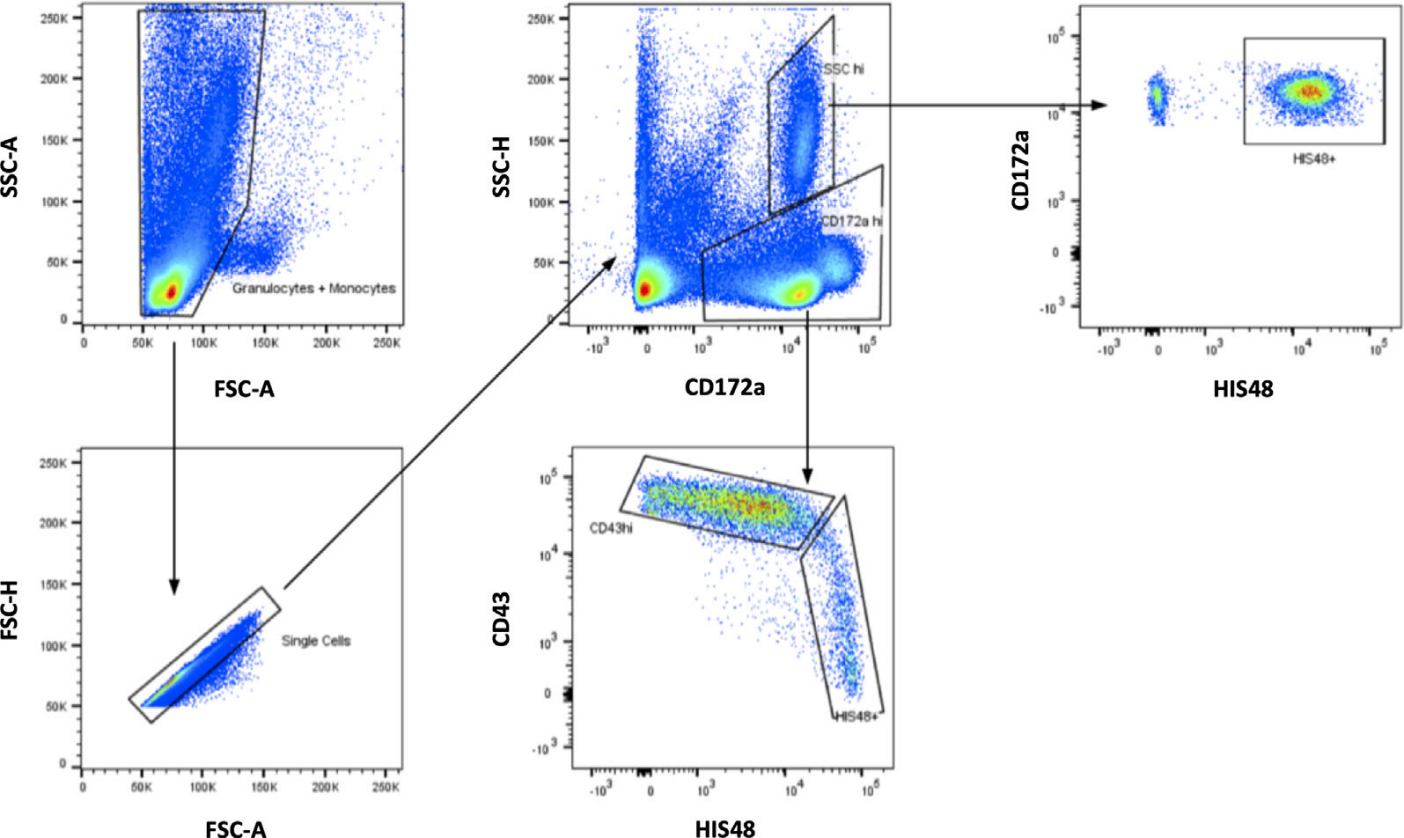
Gating strategy for rat monocytes. After initial visualization of the cells based on SSC and FSC, monocytes were detected among CD172a^+^ cells. Within this gate, smaller CD43^++^HIS48^low^ monocytes and larger CD43^low^HIS48^++^ monocytes could be separated.

We first separated monocytes from neutrophil granulocytes and lymphocytes based on cell size and internal complexity for the analysis of blood monocyte in humans. Then, we detected classical (CD14^++^ CD16^-^), intermediate (CD14^++^ CD16^+^), and non-classical (CD14^dim^ CD16^++^) monocytes among the CD14^+^ and CD16^+^ cells, which were both positive for HLA-DR and negative for B-cell (CD19^-^), T-cell (CD2^-^), NK-cell (NKp46^-^), and granulocyte (CD15^-^) markers. **Figures 1A–D** shows the detailed gating strategy for human whole blood analysis. Subsequently, we analyzed the subset-specific monocyte activation states under various conditions by assessing MAN-1 or cytokine production. In rats, we detected the two monocyte subpopulations (CD43^low^ HIS48^++^ classical monocytes and CD43^++^ HIS48^low^ non-classical monocytes) within CD172a^+^ cells, which we further characterized by measuring CD11b and intracellular staining of cytokine expression. **Figure 2** shows the detailed gating strategy for whole blood analysis in rats. We only analyzed CD14^++^ CD16^-^ (classical) and CD14^dim^ CD16^++^ (non-classical) monocytes in human samples for the presented activation assays to ensure interspecies comparability.

**Figure 3:**
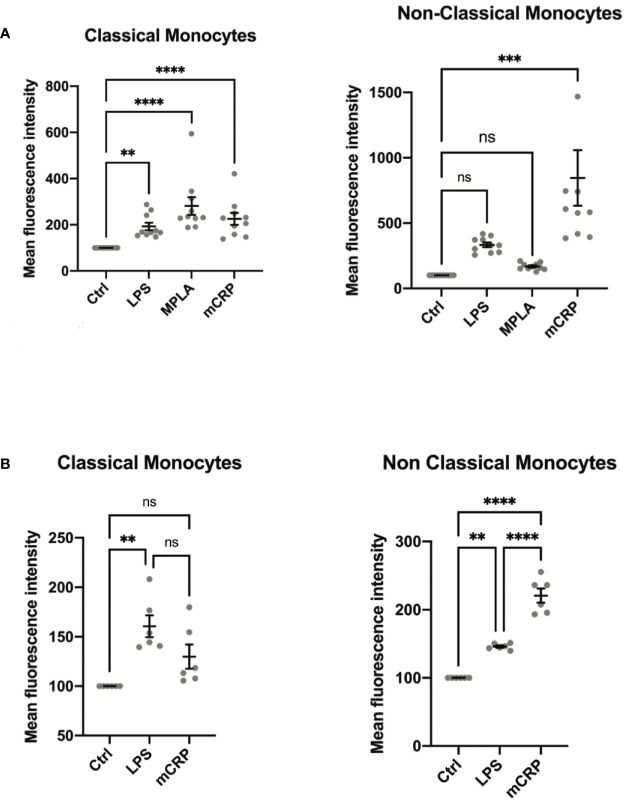
**(A)** Monocyte activation state was assessed in whole blood samples of ten healthy individuals stimulated with PBS (control), LPS, MPLA, and mCRP for 15 minutes. An increase in MAN-1 expression demonstrated cell activation. The MFI for MAN-1 of the respective control sample was set as 100. Monocyte activation levels assessed by MAN-1 expression revealed to be highly subset-specific after incubation of the cells with mCRP, whereas stimulation of monocytes with LPS and MPLA led to a general increase in monocyte activation. **(B)** Whole blood samples of six Wistar rats were analyzed after incubation with PBS (control), LPS, MPLA, and mCRP for 15 minutes, respectively. An increase in CD11b expression demonstrated cell activation. The MFI of the respective control sample was set as 100. Monocyte activation levels assessed by CD11b expression were also highly subset-specific following the pattern seen in human samples, which confirmed the interspecies transferability of the findings. ns, not significant; **p < 0.01; ***p < 0.001; ****p < 0.0001.

Monocyte activation levels assessed by MAN-1 expression revealed to be highly subset-specific after incubation of the cells with mCRP, whereas stimulation of monocytes with LPS and MPLA led to a general increase in the activation state of all monocyte subpopulations (**Figure 3A**). In analogy to our results in humans, whole blood analyses in rats showed a similar subset-specific activation pattern when stimulated with mCRP, whereas LPS incubation induced an increase of CD11b levels in both subpopulations (**Figure 3B**). These findings confirmed the interspecies transferability of our findings.

**Figure 4:**
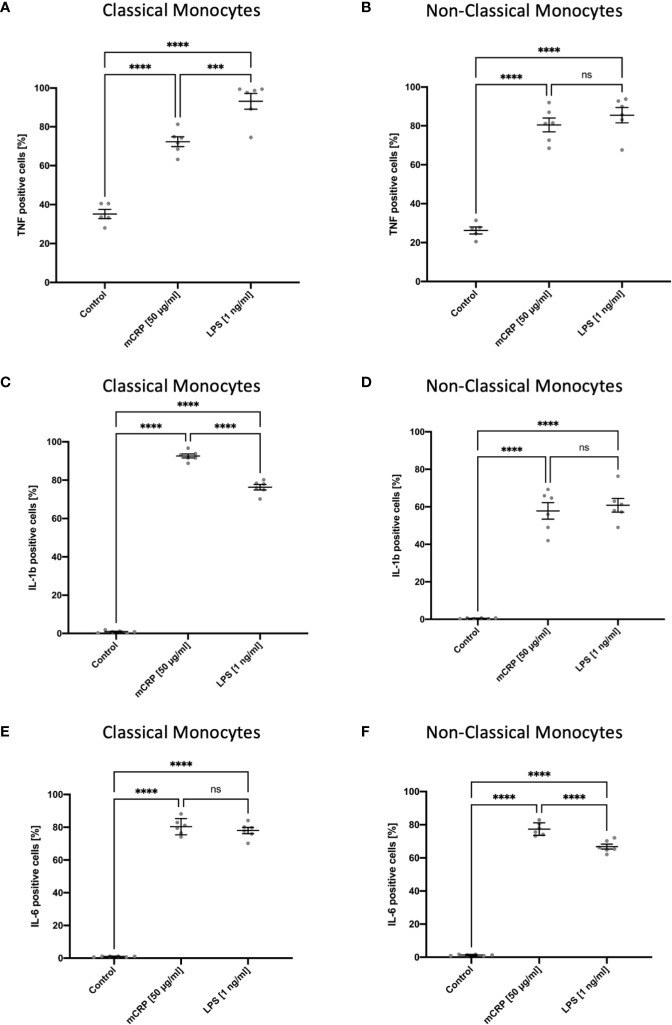
Subset-specific assessment of intracellular TNF, IL1b, and IL6 production in circulating blood monocytes after incubation in whole blood for six hours with PBS (control), mCRP, and LPS. Brefeldin A was used to inhibit the protein transport from the endoplasmic reticulum to the Golgi complex. Both monocyte subsets showed a rapid increase of TNF-positive monocytes in a subset-specific manner upon LPS and mCRP stimulation with non-classicals monocytes being the main source of TNF **(A, B)**. For IL1b, a subset-specific upregulation of intracellular production, predominantly in classical monocytes, was found both following mCRP and LPS stimulation. Within classical monocytes, IL1b production was significantly higher upon mCRP stimulation **(C, D)**. In contrast, the intracellular upregulation of IL6 production markedly increased upon both reagents and showed a subset-specific pattern only following LPS incubation **(E, F)**. ns, not significant; ***p < 0.001; ****p < 0.0001.

After incubating whole blood samples with LPS in the presence of Brefeldin A for 6 hours, both monocyte subsets showed an upregulation of TNF, which was highly subset-specific with non-classical monocytes being the primary source of TNF (**Figures 4A, B**). These findings are consistent with previous studies on TNF expression in human circulating blood monocytes (29). We also observed intracellular TNF production markedly upregulated upon mCRP stimulation in a subset-specific manner. Furthermore, we found a subset-specific upregulation pattern when analyzing the intracellular changes in IL1b production upon mCRP and LPS stimulation in human blood monocytes (**Figures 4C, D**). Here, classical monocytes proved to be the primary source of IL1b production, demonstrating a marked upregulation of the cytokine upon mCRP stimulation. In contrast, mCRP and LPS led to a comparable intracellular upregulation of IL6 production in both monocyte subsets (**Figures 4E, F**). However, for non-classical monocytes, intracellular IL6 production was significantly higher upon mCRP stimulation, whereas there was no difference between the two reagents in classical monocytes. Again, these findings are comparable with previously published functional analyses of monocyte subsets (16). The evaluation of cytokine expression in MACS-enriched monocyte suspensions upon LPS stimulation using RT-PCR lacked a subset-specific differentiation of RNA levels. Thus, no correlation could be drawn to the intracellular staining assay (**Supplemental Information 3**).

Furthermore, we found non-classical monocytes to lose the CD16 surface marker almost entirely after the extended incubation period for cytokine analysis, thus being non-detectable by the gating strategy mentioned above. However, we could readily identify non-classical monocytes by backtracking the cells within the HLA-DR^+^ gate (**Figures 1E–G**). Interestingly, CD16 expression remained stable and detectable in monocytes when incubated with PBS for 6 hours (control samples).

**Figure 5:**
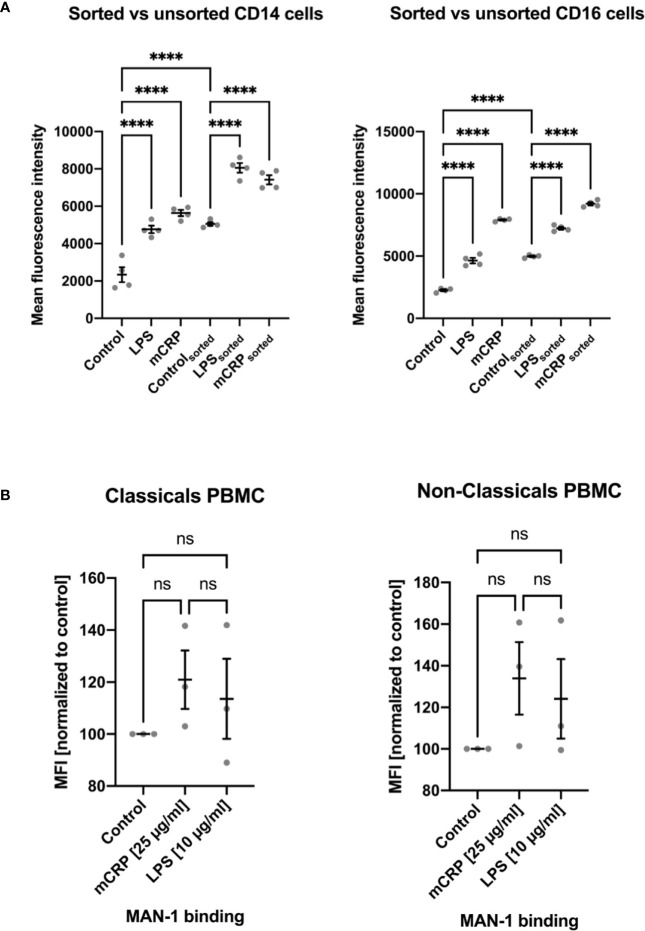
Comparison of monocyte activation state in whole blood samples upon stimulation with PBS (control), mCRP, and LPS to activation states assessed after cell isolation *via* fluorescence-activated cell sorting **(**FACS; **A)** and density gradient centrifugation (PBMC; B). An increase in MAN-1 expression demonstrated cell activation. The MFI for MAN-1 of the respective control sample was set as 100. Both monocyte subtypes showed a marked upregulation of MAN-1 after cell isolation itself, especially following density gradient centrifugation **(B)**. While FACS led to a moderate increase in cell activity, allowing the evaluation of monocytes’ activation state upon further stimulation **(A)**, PBMC isolation resulted in an out of scale pre-activation of the cells that rendered further stimulation insignificant **(B)**. ns, not significant; ****p < 0.0001.

Since surface contact or a temperature change can alter monocytes’ activation state, we found cell fixation immediately after incubation with an activating reactant to be crucial to analyze the reagent-dependent upregulation of integrins and cytokines. Therefore, any following processing step after cell fixation, e.g., osmotic lysis and cell staining, did not further increase activation levels or cytokine expression. We observed a markedly increased activation of monocyte subsets after PBMC isolation *via* density gradient centrifugation and, less emphasized, following fluorescence-activated cell sorting. MAN-1 expression proved to be significantly higher after cell isolation than observed in the whole blood assays (**Figure 5**, **Supplemental Information 4**). While the moderate pre-activation of monocyte subsets following FACS still allowed to distinguish subset-specific patterns upon different stimulating reagents (**Figure 5A**), PBMC isolation led to a disproportionate activation rendering further stimulation of the cells insignificant (**Figure 5B** and **Supplemental Information 4**).

**Figure 6:**
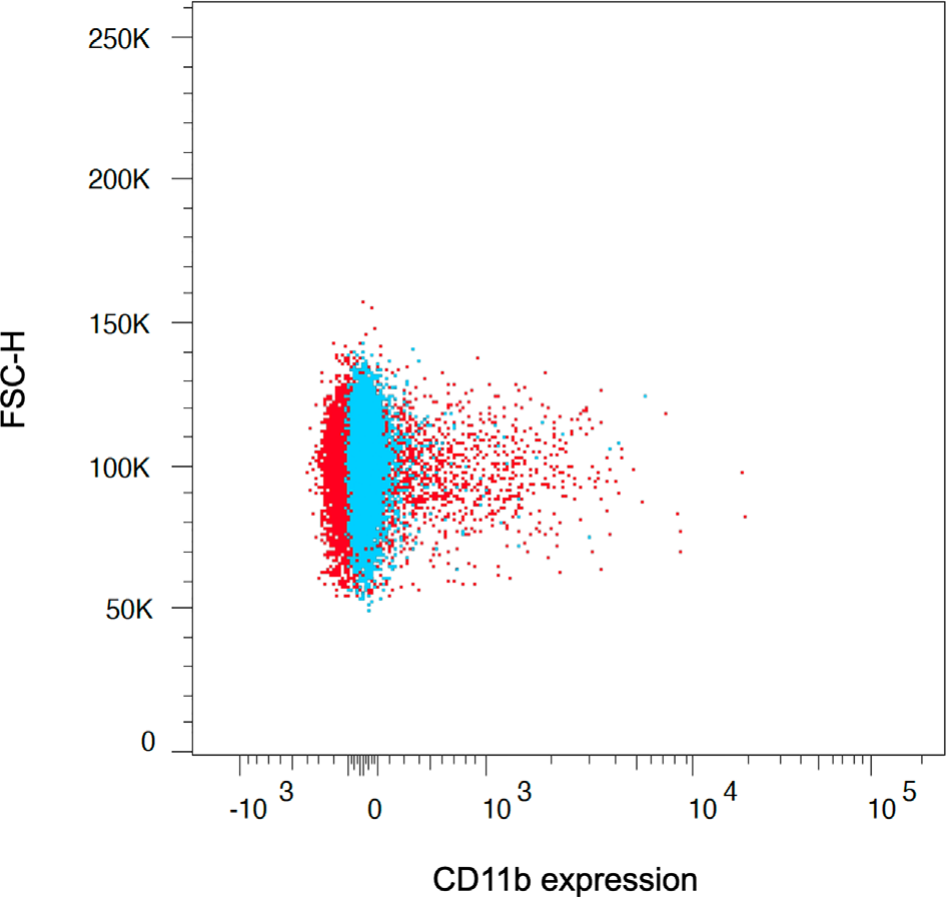
The application of ready-to-use lysis solutions, which combine red cell lysis and cell fixation at the same time, led to unpurified and more activated monocyte subsets due to further stimulation of the cells (red population). Therefore, separation of cell fixation and red cell lysis is crucial to assess the reagent-dependent upregulation of integrins and cytokines, which we achieved with the presented lysis buffer and protocol (blue population). The graph depicts the CD11b expression on CD14++ classical monocytes after red cell lysis and fixation using ready-to-use lysis solution (red population) and facilitating the presented approach (blue population).

Furthermore, we used an ammonium chloride-based lysis solution for both human and rat red blood cells, which, in contrast to many commercially available lysis solutions, does not include a cell fixation reagent. We observed more extended osmotic lysis periods and incomplete red cell lysis in our experiments when using ready-to-use lysis solutions. Thus, monocytes tended to be further stimulated, resulting in increased metabolic activation states (**Figure 6**). This is especially true for rat monocytes, which are more fragile and susceptible to osmotic alteration (21, 30).

## Discussion

Our study presents a comprehensive flow cytometry-based approach to assess subset-specific activation and cytokine expression of circulating blood monocytes both in humans and rats. To accurately evaluate differential monocyte regulation without the cell isolation process’s interference, we strategically separated cell activation, cell fixation, and subsequent red blood cell lysis. Since surface expression markers on monocytes can change depending on their activation state, it is of utmost importance to apply robust flow cytometry gating techniques to detect monocyte subpopulation under various conditions. Therefore, we developed a novel gating strategy by incorporating previously described inclusion and exclusion gating techniques (31).

We found a differential CRP-mediated activation of monocyte subsets in both human and rat blood samples by utilizing the presented methodological approach. In contrast, short-term incubation with LPS and MPLA unspecifically increased the activation state of all monocytes. Furthermore, both monocytic subsets showed an intracellular upregulation of TNF after incubation with LPS and mCRP. Here, we found non-classical monocytes to be the main source of TNF. We observed a homologous cytokine expression pattern in rat monocytes, which confirmed our method’s interspecies transferability. For the leukocytic pyrogen IL1b, we also found a subset-specific upregulation of intracellular production, predominantly in classical monocytes, both following mCRP and LPS stimulation. Within classical monocytes, IL1b production was significantly higher upon mCRP stimulation. In contrast, the intracellular upregulation of IL6 production markedly increased upon stimulation with both reagents and showed a subset-specific pattern following LPS incubation. Again, these findings are consistent with previous studies on functional analyses of monocyte subsets (16, 29). The presented results demonstrated the assays’ feasibility to accurately identify subset-specific differences in monocyte behavior, both in human and rat blood samples. This is a crucial advantage over functional assays using purified or enriched cell suspensions, which lack subset-specific assessment of cytokine regulation.

The following two steps are crucial to ensure accurate monocyte subset analysis and avoid confounding of experimental results. Firstly, cells need to be fixed after the stimulation step to stop further stimulation. Secondly, the fixed cell solution needs to be washed before performing red blood cell lysis to ensure rapid and gentle lysis. Thirdly, we recommend temporally separating cell fixation and red blood cell lysis to virtually leave monocytes’ activation state untouched and allow for a non-confounded monocyte analysis. Most commercially available lysis buffers contain a fixation reagent, thus combining both steps and presumably leading to a significantly higher monocyte activation state. The lysis buffer presented in our protocols consists of commonly available reagents and can be prepared as a cost-effective, highly concentrated stock solution. We freshly prepare working solutions for best results and adjust them to species-specific pH-levels once a week.

Circulating blood monocytes, which usually do not include other monocyte subsets that are marginated, i.e., patrolling monocytes on endothelial vasculature, unfold considerable heterogeneity regarding their phenotype, transcriptional profile, and function (1, 32, 33). Given their role in the pathogenesis of various diseases, subset-specific characterization of blood monocytes has been the focus of several investigations (16, 34–39). However, most studies have relied on isolating monocytes from PBMCs by labeling surface antigens or density gradient centrifugation, which both can induce changes in the distribution and activity of monocytic subsets (17, 18). Our analysis confirmed that both PBMC isolation and fluorescence-activated sorting resulted in a pre-activation of monocyte subsets, thereby confounding further stimulation of the cells and downstream applications. Therefore, we suggest investigating cell activity and cytokine expression of human and rat monocyte subsets directly in whole blood samples. In our approach, we evaluated and optimized the osmotic lysis of red blood cells and antibody staining to accurately assess the subset-specific cell activation state without further alteration of cell metabolism.

The *in vitro* results may approximate monocytes’ physiological behavior since we stimulated and stained the cells in whole blood. However, the *in vitro* approach fails to provide information about the molecular mechanism of cell activation involved. Thus, the flow cytometry-based analysis does not reveal whether the specific reagent alone is directly responsible for inducing cytokine upregulation in monocytes, which is a limitation of the presented protocol. The whole blood analysis only reflects intracellular cytokine production due to brefeldin A inhibiting the protein transport from the endoplasmic reticulum to the Golgi complex. As cytokines can be post-translationally modified during maturation, this approach is limited to the antibody recognition of non-maturated cytokines. However, it still allows distinguishing subset-specific differences in cytokine production.

Our method facilitates an endpoint analysis of cell activity under physiological circumstances, but it does not allow for downstream applications, which is an explicit limitation of the protocol. In this context, the methodological approach is a valuable addition to existing monocyte analysis protocols that involve cell isolation and further cell culture evaluation. Therefore, we recommend utilizing the presented method to analyze subset-specific monocyte behavior in (animal) disease models. As small blood samples (50 - 100 μL) are sufficient to carry out the analysis, repeated analyses in the same subject are feasible and, thus, allow for investigating the role of monocyte subsets in various clinical studies and animal disease models.

Pre-analytic sample preparation in functional assays may pose other problems. For example, magnetic-activated cell sorting (MACS) is a commonly used method for purification or enrichment purposes, either based on the specific expression (positive sorting) or the lack of surface markers (negative cell sorting) in the cell population of interest. Thus, the expression of cell surface markers has to differ significantly to allow the separation of cells. Therefore, magnetic cell sorting might not be sensitive enough for the differential expression of CD14 and CD16 on monocyte subsets, which impedes subset-specific cell separation. Secondly, the fitting antibody-conjugated microbeads have to be provided. Thirdly, even though beads for magnetic cell sorting are designed to be non-toxic and do not affect the activation state of the cells, it has been shown that positively sorted CD14^+^ monocytes, as opposed to negatively sorted ones, exhibit reduced activation and proliferation capacity after stimulation with LPS (40). Therefore, MACS can introduce a bias in downstream analyses for both activation assays and interference with immunofluorescent staining.

In conclusion, the above experiments demonstrated that the protocols presented herein are reproducible and valid to accurately assess the subset-specific monocyte activation state in an endpoint analysis. Moreover, the method provides insight into subset-specific monocyte activities in biological contexts that elicit an inflammatory state, such as cardiovascular diseases, viral and bacterial infections, and allogeneic immune response.

## Data Availability Statement

The original contributions presented in the study are included in the article/**Supplementary Material**. Further inquiries can be directed to the corresponding author.

## Ethics Statement

The studies involving human participants were reviewed and approved by Ethics Committee of the University of Freiburg (Ethikkommission der Albert-Ludwigs-Universität Freiburg). The patients/participants provided their written informed consent to participate in this study. The animal study was reviewed and approved by Regional Commission for Veterinary Foodstuff Control Freiburg (Regierungspräsidum Freiburg, Veterinärwesen & Lebensmittelüberwachung).

## Author Contributions

Concept and design: JK, JZ, and SE. Conception of experimental design: JZ. Acquisition, analysis, and interpretation of the data: all authors. Drafting of the manuscript: JK. Critical revision of the manuscript for intellectual content: all authors. Supervision: SE. All authors contributed to the article and approved the submitted version.

## Funding

This work was supported by personal grants to SE from the German Research Foundation (DFG) EI 866/1-1, EI 866/1-2, EI 866/5-1, and EI 866/10-1. SE is a Heisenberg Professor of the DFG (EI 866/4-1 and EI 866/9-1). 

The article processing charge was funded by the Baden-Wuerttemberg Ministry of Science, Research and Art and the University of Freiburg in the funding programme Open Access Publishing.

## Conflict of Interest

The authors declare that the research was conducted in the absence of any commercial or financial relationships that could be construed as a potential conflict of interest.

## Supplementary Material

Supplemental Information 1 Phenotyping, activation state, and cytokine assessment protocol for human monocytes.

Supplemental Information 2 Phenotyping, activation state, and cytokine assessment protocol for rat monocytes.

Supplemental Information 3 Functional analysis of cytokine expression in monocytes using RT-PCR. To compare monocytic cytokine expression assessed by RT-PCR to intracellular cytokine staining, we first purified monocytes from PBMC using magnetic-activated cell sorting (MACS; Pan Monocyte Isolation Kit human, Miltenyi Biotec). We then incubated the monocytic cell suspension with PBS (control vehicle) and LPS (10 ng/mL and 100 ng/mL) in 0.5% PBS-BSA for 3, 6, and 24 h. After RNA isolation, we accessed the expression of TNF using RT-PCR. TNF expression continuously increased upon stimulation with 10 and 100 ng/mL LPS over time. After 3 hours, we observed no significant increase in TNF expression upon LPS stimulation compared to PBS. The TNF expression peaked after 6 hours for both LPS concentrations with the higher concentration leading to significantly higher gene expression. Further incubation did not increase TNF production. However, this approach did not allow for a subset-specific differentiation of cytokine expression. Thus, no correlation could be drawn to the results of intracellular cytokine staining. ns, not significant.

Supplemental Information 4 Monocyte activation after cell isolation *via* density gradient centrifugation. In addition to **Figure 5B**, the absolute MFI values are depicted to emphasize the out of scale pre-activation of monocytes following PBMC isolation that rendered further stimulation of the cells inconclusive. ns, not significant; ***p < 0.001; ****p < 0.0001.
