# Supplementary material for: An Unbiased Flow Cytometry-Based Approach to Assess Subset-Specific Circulating Monocyte Activation and Cytokine Profile in Whole Blood
Source: Front Immunol. 2021 Apr 26;12:641224. doi: 10.3389/fimmu.2021.641224 (PMC8108699; doi:10.3389/fimmu.2021.641224)
Supplement: Supplemental Information 1 — Phenotyping, activation state, and cytokine assessment protocol for human monocytes. [file DataSheet_1.docx]

**Phenotyping and assessing activation state of human monocytes from whole blood**

1. **Buffers and materials**

RBC lysis buffer (10x): 80.2 g NH_4_Cl

8.4 g NaHCO_3_

126 ml EDTA (100 mM)

1000 ml total end volume, fill up with milliQ water

- prepare 1x dilution, preferably 500 ml, and autoclave it before use; keep buffer solution at 4°C (pH 7.8)
- renew buffers every week

FACS-buffer:

PBS-BSA (0.5 %) 5 g BSA (Sigma: A9418-100G)

1l sterile PBS

- filtered with 0.45 µm and stored at 4°C

Fixation-buffer:

10x BD CellFix

- prepare 1x Solution with ddH_2_O and store at room temperature

Antibodies:

anti-HLA-DR-FITC (TU36, mouse IgG2b, κ, BD Biosciences) 1:50

anti-CD14-PacB (M5E2, mouse IgG2a, κ, BD Biosciences) 1:50

anti-CD16-PE-Cy7 (3G8, mouse IgG1, κ, BD Biosciences) 1:50

Negative lineage markers:

anti-CD2-PE (RPA-2.10, mouse IgG1, κ, BD Biosciences) 1:50

anti-CD19-PE (HIB19, mouse IgG1, κ, BD Biosciences) 1:50

anti-CD15-PE (VIMC6, mouse IgM, κ, BD Biosciences) 1:50

anti-CD56-PE (MY31, mouse IgG2, κ, BD Biosciences) 1:50

anti-NKp46-PE (BAB281, mouse IgG1, κ, BD Biosciences) 1:50

1. **Procedure**

Volumes can be adjusted if necessary, but the ratio should be kept as indicated.

1. **Cell stimulation**
2. Transfer 100 µl of freshly taken whole blood (heparinized) for each sample into 2 ml polypropylene collection tubes (Biozym Scientific GmbH)
3. Stimulate samples for 15 min at 37°C, 5% CO_2_ (incubator) with reagents
4. Add 2.4 µl MAN-1 (or 2µl CD11b) and stain for 15 min on ice and in the dark. For MAN-1 analysis, add 2.0 µl Penta-His 647 (APC) for MAN-1 afterward and incubate for 10 min on ice and in the dark
5. Add 50 µl of 1x CellFix dilution to each sample to stop cell activation and fix the cells for 5 min at room temperature
6. **RBC lysis**
7. Perform osmotic lysis of red blood cells adding freshly prepared lysis buffer (4°C) using a ratio of 1:20 v/v
   - spin at 200x g for 7 min at 4°C
   - mix the solution and transfer half of it into a 2^nd^ polypropylene collection tube, resuspend and fill up both tubes with fresh lysis buffer (1:20 v/v)
8. Incubate for 5 min at 4°C and spin at 200x g for 7 min at 4°C
   - take off the supernatant and resuspend both tubes in 400 µl RBC lysis buffer
9. Incubate 2 min at 4°C; fill up with PBS++ to 500 µl and spin at 200x g for 7 min at 4°C
10. Take off the supernatant, wash pellets with cold PBS++ (250 µl) and spin at 120x g for 15 min at 4°C
11. Take off the supernatant, unite pellets in one tube and resuspend pellet in 400 µl of PBS++ total volume
12. Spin remaining cells at 150x g for 8 min at 4°C (required concentration: 10^6^ cells in 50 µl = 2x10^7^ / ml)
13. **Cell staining**
14. Take off the supernatant and resuspend in 100 µl of FACS-buffer
15. Stain cells with antibodies and incubate for 15 min at 4°C in the dark
16. Add 150 µl of FACS buffer and spin at 480x g for 5 min at 4°C
17. Take off the supernatant, add 50 µl of 4% PFA in PBS (for analysing within the next 24 hours), add 350 µl of FACS buffer and transfer to 5 ml FACS tubes (polystyrene) for immediate analysis (end volume: 500 µl)
