# Supplementary material for: An Unbiased Flow Cytometry-Based Approach to Assess Subset-Specific Circulating Monocyte Activation and Cytokine Profile in Whole Blood
Source: Front Immunol. 2021 Apr 26;12:641224. doi: 10.3389/fimmu.2021.641224 (PMC8108699; doi:10.3389/fimmu.2021.641224)
Supplement: Supplemental Information 2 — Phenotyping, activation state, and cytokine assessment protocol for rat monocytes. [file DataSheet_2.docx]

**Phenotyping and assessing activation state of rat monocytes from whole blood**

1. **Buffers and materials**

RBC Lysis buffer:

8.3g NH_4_Cl

1g NaHCO_3_

1ml EDTA (100mM)

1000 ml total end volume, fill up with milliQ water

- prepare 1x dilution, preferably 500 ml, and autoclave it before use; keep buffer at 4°C (pH 7.7)
- renew buffers every week

FACS-buffer:

PBS-BSA (0.5 %) 5 g BSA (Sigma; A9418-100G)

1l sterile PBS

- filtered with 0.45 μm and stored at 4°C

Fixation-buffer:

10x BD CellFix

- prepare 1x Solution with ddH_2_O and store at room temperature

Antibodies:

anti-CD172A FITC (OX41 Clone, BIO-RAD #MCA274F) 1:40

anti-CD43 APC (W3/13, BioLegend #202810) 1:40

anti-HIS48 Biotin (HIS48, eBioscience #13-0570-82) 1:40

Streptavidin eFluor 450 (eBioscience #48-4317-82) 1:40

Anti-CD11b PE (WT.5, BD Pharmingen #562105) 1:40

1. **Procedure**

Volumes can be adjusted if necessary, but the ratio should be kept as indicated.

1. **Cell stimulation**
2. Transfer 100 µl of freshly taken whole blood (heparinized) for each sample into 15 ml polypropylene collection tubes (Biozym Scientific GmbH)
3. Stimulate samples for 15 min at 37°C, 5% CO_2_ (incubator) with reagents
4. Add 2.0 µl CD11b rat antibody and stain for 10 min on ice and in the dark
5. Add 50 µl of 1x CellFix dilution to each sample to stop cell activation and fix the cells for 5 min at room temperature
6. **RBC lysis**
7. Perform osmotic lysis of red blood cells adding freshly prepared lysis buffer (4°C) using a ratio of 1:20 v/v (add no more than 1 ml of blood to 5 ml of RBC lysis buffer)
8. Incubate for 4 min on ice and then add 5ml of PBS**-**BSA 0.5 % (FACS-buffer)
9. Spin at 480x g for 6 min at 4°C
10. Carefully discard the supernatant, removing as much liquid as possible without disturbing the pellet
11. Resuspend the pellet in 200 µl of PBS-BSA 0.5 % and transfer to round bottom 96 well plate (non-tissue culture treated) or polypropylene FACS tubes
12. Spin at 480x g for 5 min at 4°C
13. **Cell staining**
14. Resuspend in 100 µl PBS-BSA (0.5 %), add 3.6 µl of antibody mixture (CD172a, CD43, HIS48 Biotin) and stain for 15 min on ice (all dilution 1/40)
15. Complete to 200 µl with PBS-BSA (0.5 %) and spin at 480x g for 5 min at 4°C
16. Discard the supernatant, resuspend in 200 µl PBS-BSA (0.5 %), add 1.0 µl of Streptavidin-Pacific blue (dilution 1/200) and stain for 20 min on ice
17. Spin at 480x g for 5 min at 4°C
18. Discard the supernatant, resuspend in 350 μl PBS-BSA (0.5 %), add 50 µl of 4 % PFA in PBS for a total volume of 400 μl and transfer to polystyrene FACS tube for analysis *(4 % PFA to be made up fresh or defrosted and then kept in fridge to be used within 1 week)*
