# Supplementary figures and images for: An Unbiased Flow Cytometry-Based Approach to Assess Subset-Specific Circulating Monocyte Activation and Cytokine Profile in Whole Blood
Source: Front Immunol. 2021 Apr 26;12:641224. doi: 10.3389/fimmu.2021.641224 (PMC8108699; doi:10.3389/fimmu.2021.641224)

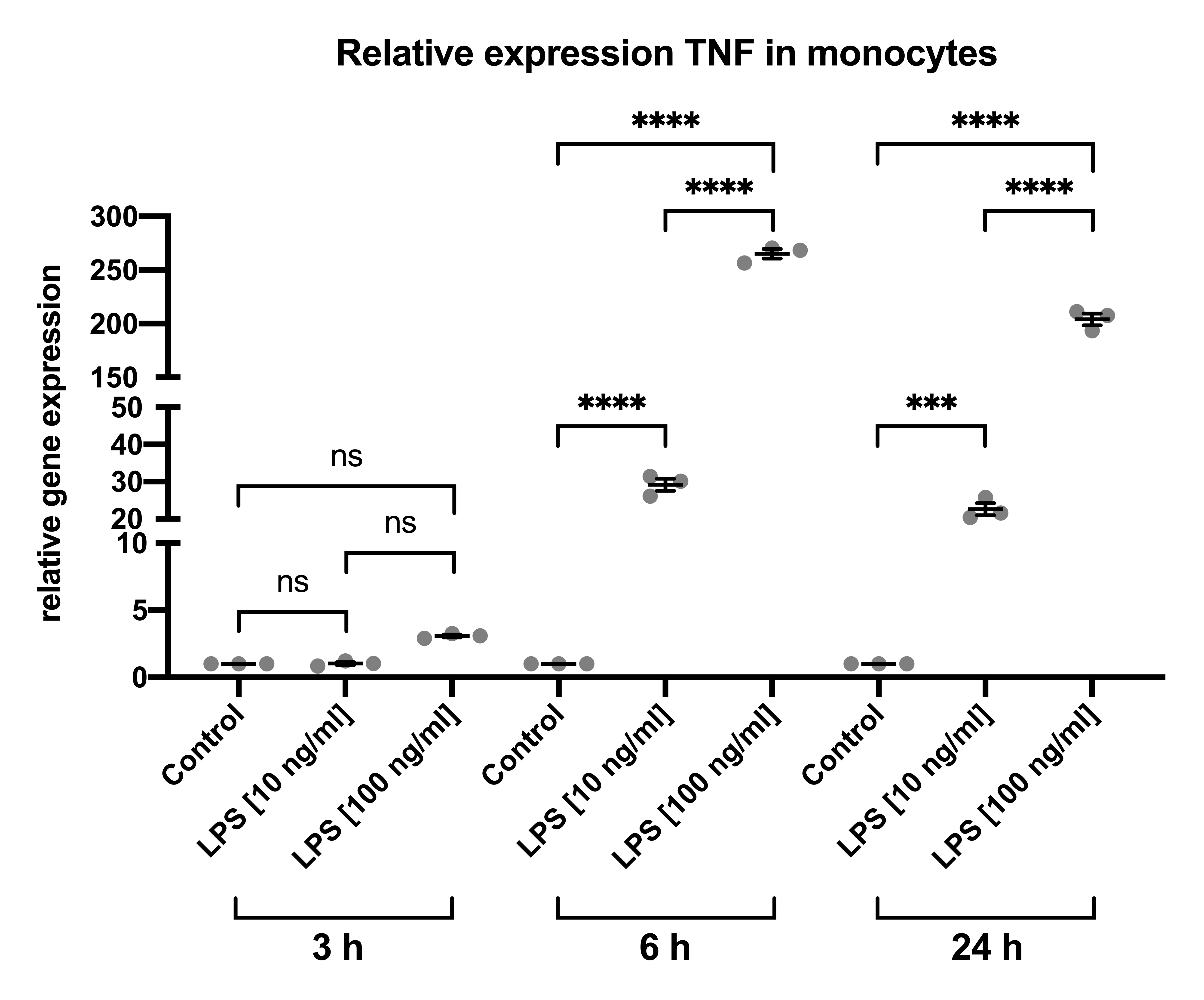

Supplement: Supplemental Information 3 — Functional analysis of cytokine expression in monocytes using RT-PCR. To compare monocytic cytokine expression assessed by RT-PCR to intracellular cytokine staining, we first purified monocytes from PBMC using magnetic-activated cell sorting (MACS; Pan Monocyte Isolation Kit human, Miltenyi Biotec). We then incubated the monocytic cell suspension with PBS (control vehicle) and LPS (10 ng/mL and 100 ng/mL) in 0.5% PBS-BSA for 3, 6, and 24 h. After RNA isolation, we accessed the expression of TNF using RT-PCR. TNF expression continuously increased upon stimulation with 10 and 100 ng/mL LPS over time. After 3 hours, we observed no significant increase in TNF expression upon LPS stimulation compared to PBS. The TNF expression peaked after 6 hours for both LPS concentrations with the higher concentration leading to significantly higher gene expression. Further incubation did not increase TNF production. However, this approach did not allow for a subset-specific differentiation of cytokine expression. Thus, no correlation could be drawn to the results of intracellular cytokine staining. ns, not significant. [file Image_1.tiff]

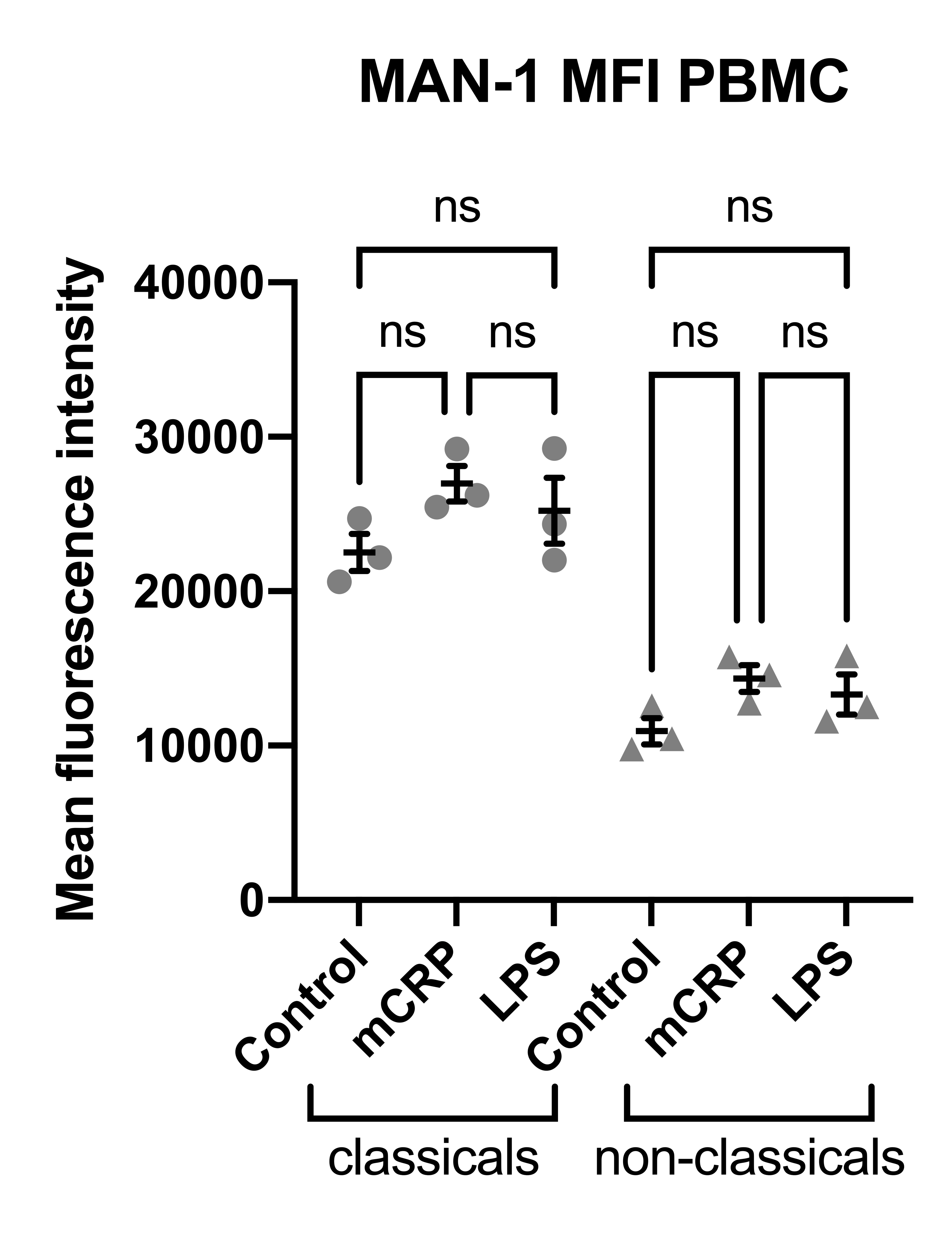

Supplement: Supplemental Information 4 — Monocyte activation after cell isolation via density gradient centrifugation. In addition to Figure 5B , the absolute MFI values are depicted to emphasize the out of scale pre-activation of monocytes following PBMC isolation that rendered further stimulation of the cells inconclusive. ns, not significant; ***p < 0.001; ****p < 0.0001. [file Image_2.tiff]
